# Variant form of hairy cell leukemia

**DOI:** 10.1002/ccr3.2176

**Published:** 2019-05-06

**Authors:** Margaux Wiber, Elsa Maitre, Edouard Cornet, Véronique Salaün, Dina Naguib, Xavier Troussard

**Affiliations:** ^1^ Laboratoire d’Hématologie CHU Caen Caen France; ^2^ Normandie Université, INSERM U1245 Université de Caen Caen France; ^3^ Institut d'Hématologie de Basse‐Normandie CHU Caen Caen France

**Keywords:** *BRAF*^V600E^, chronic B‐lymphoproliferative disorder, hairy cell leukemia, hairy cell leukemia variant, Ibrutinib, KDM6A

## Abstract

Mature lymphoid B‐cell proliferations with hairy cells represent heterogeneous entities where specific diagnosis is difficult but important since it impacts therapeutic management. The clinical cases of variant hairy cell leukemia reported herein illustrate the persistence of a clear interest in the use of splenectomy as a therapeutic alternative. Furthermore, ibrutinib appears to be a promising treatment in patients with relapsed/refractory disease.

## 1 INTRODUCTION

The variant form of hairy cell leukemia (vHCL) is a rare B‐cell chronic lymphoid disorder and is considered as a provisional entity in the most recent revision of the WHO 2016 classification.^1^ vHCL differs from the classical form of the disease (cHCL) by morphological, phenotypic, and molecular criteria. In vHCL, the abnormal lymphoid cells do not usually express CD25 and CD123.^2^ Unlike cHCL, the *BRAF^V600E^* gene mutation is not present in vHCL. In contrast, *MAP2K1* gene mutations are identified in about one‐third of vHCL cases.^3^ It is important to distinguish these two entities, since vHCL has a more aggressive clinical presentation with no response to purine analogs. Unlike cHCL, new therapeutic approaches^4^ have to be validated in vHCL.

In view of the presence of hairy cells on examination of the blood smear, it is also worth mentioning the diagnosis of splenic lymphoma of the marginal zone (SMZL) and diffuse small B‐cell lymphoma of the splenic red pulp (SDRPL).^5^ In the absence of histological examination of the spleen, distinguishing all these entities may be difficult but necessary, given that different clinical management is required.

We report two cases of patients with vHCL presenting the *KDM6A* gene mutation^6^ and a different clinical profile.

## 2 CASE 1

**Figure 1:**
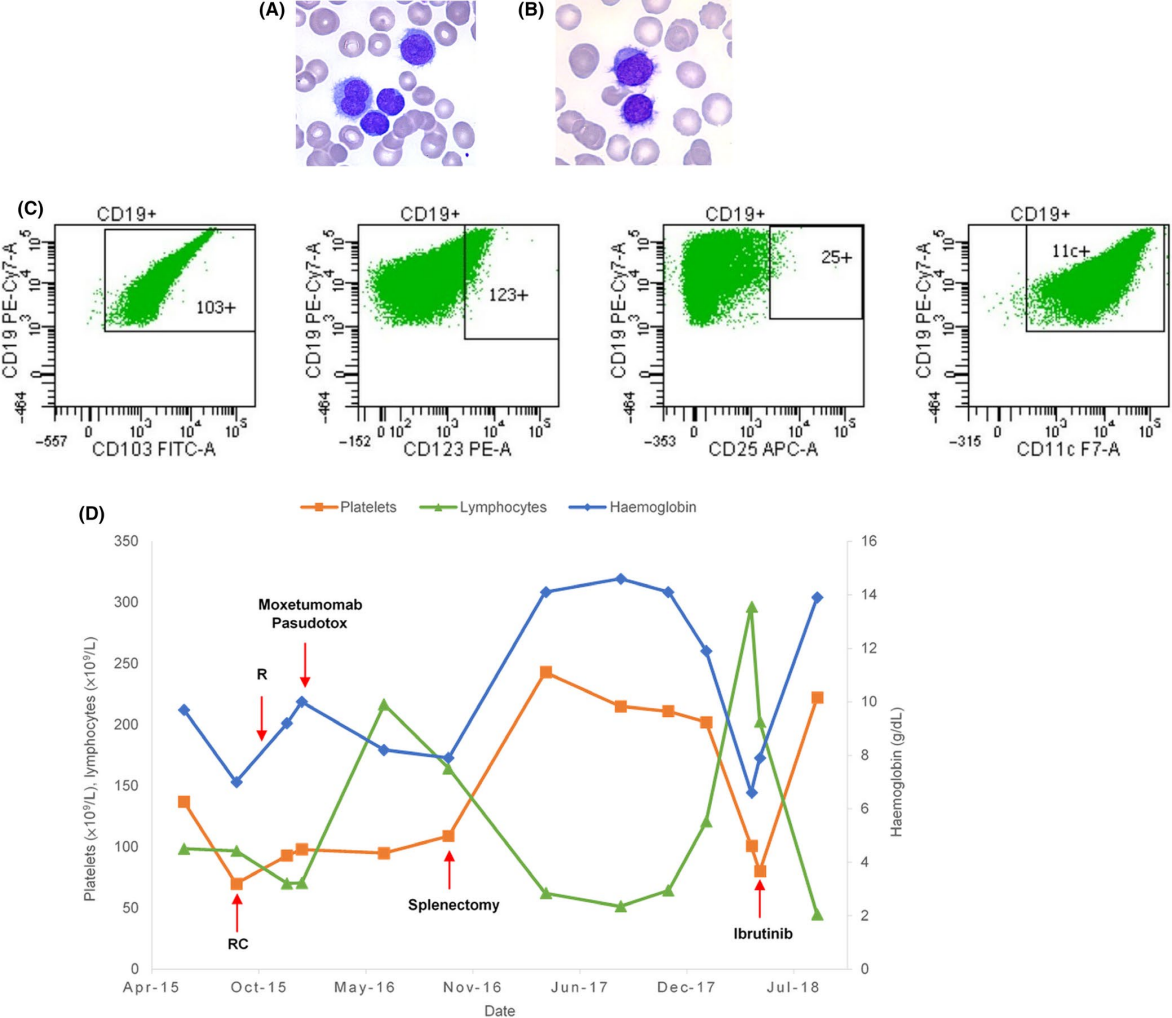
Case 1. A, Hairy cells in peripheral blood. B, Hairy cells in bone marrow. C, Flow cytometric analysis of expression levels of CD103, CD123, CD25, and CD11c (HCL score) on hairy cells. D, Complete blood count evolution. C, cladribine; R, rituximab

A 64‐year‐old patient presented with a diagnosis of vHCL. The patient's history included high blood pressure, high cholesterol, and smoking at 40 pack‐years. On clinical examination, there was a large splenomegaly, edema of the lower limbs, and dyspnoea on exertion. The thoracoabdominopelvic CT showed a heterogeneous spleen of 23 cm. The pulmonary parenchyma was the site of abnormalities, suggestive of interstitial cystic pneumopathy. There was anemia with hemoglobin 9.7 g/dL, thrombocytopenia with platelets 137 × 10^9^/L, and high leukocytosis at 106 × 10^9^/L. The lymphocytes, accounting for 93% of the leukocytes, were atypical lymphocytes of medium size, with a large and regular nucleus, sometimes bilobed, with mature chromatin and nucleolae and cytoplasm with non‐polar villi (Figure 1A). Bone marrow was infiltrated by 47% of abnormal cells (Figure 1B). The abnormal lymphoid blood cells expressed B‐cell markers, CD19, FMC7, CD20, CD79b, lambda monotypic light chain, CD11c, and CD103, without expressing CD25 and CD123 that are usually identified in cHCL (Figure 1C). The lymphoid cells were also detected in the bronchoalveolar lavage fluid. The karyotype of the peripheral blood lymphocytes showed a 7q deletion in position 22‐36, a recurrent but unspecific abnormality in vHCL. The *BRAF^V600E^* mutation was not present, confirmed by high‐throughput sequencing. A mutation in the *KDM6A* gene, encoding a protein implicated in epigenetics, as well as a subclonal mutation of the *MAP2K1* gene, was identified.^6^ The allelic frequency of this last mutation increased over time. Three months after diagnosis, treatment with cladribine (0.14 mg/kg SC J1‐J5) combined with rituximab (375 mg/m^2^ IV on day 1) was started. The treatment was not effective, and a second‐line treatment with moxetumomab pasudotox, an anti‐CD22 antibody coupled to an immunotoxin, was started according to a schedule of administration on D1, D3, D5 every 28 days for six cycles (2.9 mg IV). The treatment was again not effective. A splenectomy was performed: the spleen histological examination showed a massive lymphoid infiltration with CD20 and Bcl2 positive cells, expansion of the red pulp and disappearance of the white splenic pulp. All these features are compatible with vHCL diagnosis. Splenectomy allowed the normalization of hemoglobin and platelet counts. Lymphocytosis remained stable approximately 50 × 10^9^/L. After 18 months, the patient progressed, with an increase in dyspnoea that required oxygen therapy. The hemogram showed anemia at 6.6 g/dL and thrombocytopenia at 10 × 10^9^/L. Treatment with ibrutinib at a dose of 420 mg per day was started. Four months later, oxygen therapy was stopped; at this time, cytopenias were corrected, and leukocytosis had decreased from 200 × 10^9^/L to 45 × 10^9^/L (Figure 1D).

## 3 CASE 2

**Figure 2:**
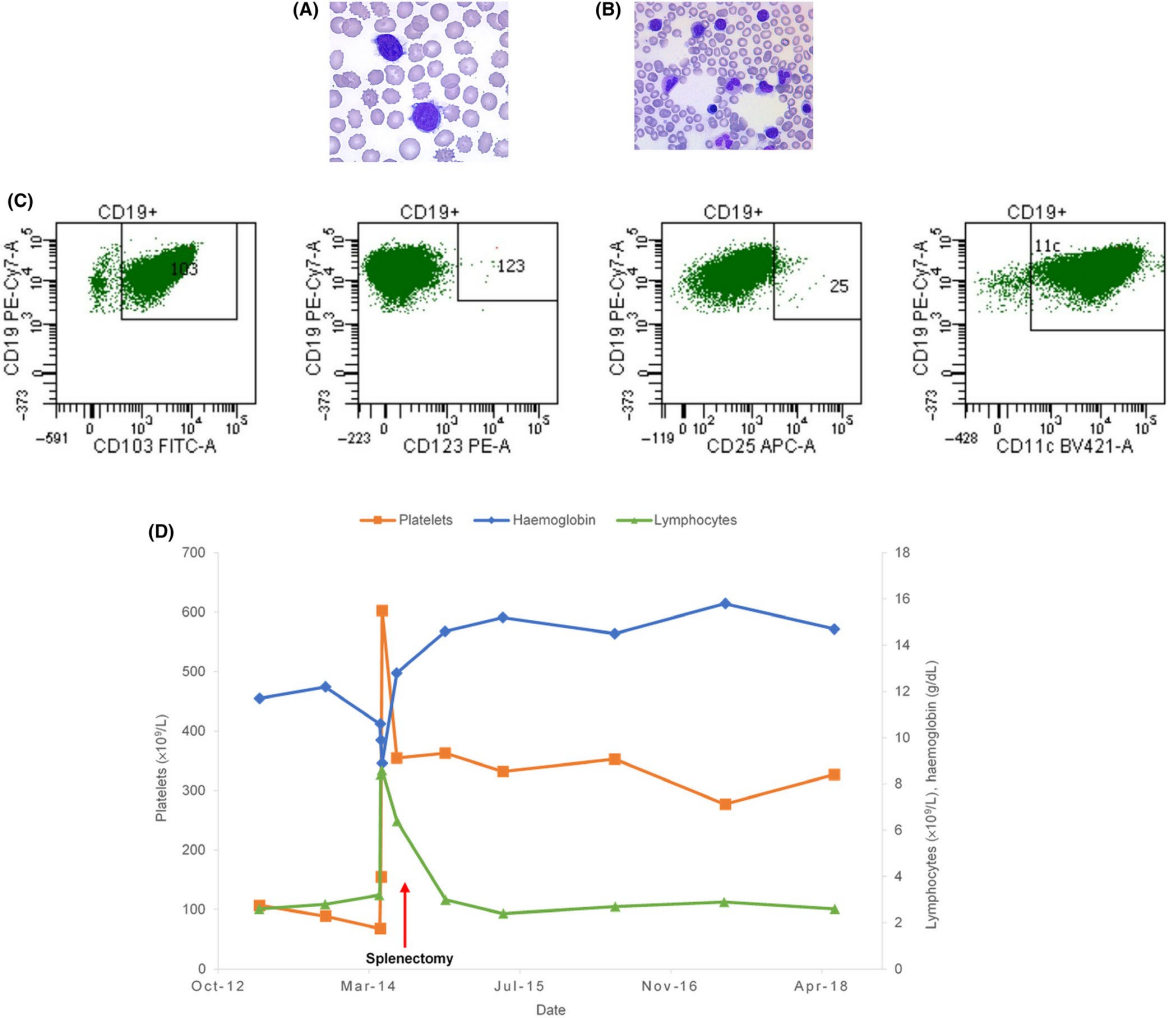
Case 2. A, Hairy cells in peripheral blood. B, Hairy cells in bone marrow. C, Flow cytometric analysis of expression levels of CD103, CD123, CD25, and CD11c (HCL score) on hairy cells. D, Complete blood count evolution

Hairy cell leukemia was diagnosed in a 72‐year‐old male with no particular antecedent. There was bone marrow infiltration (15%) by lymphoid cells expressing B‐cell markers CD19, FMC7, CD20, and CD79b, as well as a monotypic kappa light chain and the CD11c and CD103 without expressing CD25 and CD123 (Figure 2C). One year after diagnosis, treatment with cladribine for 5 days was started, but the splenomegaly remained bulky. The hemogram showed a moderate anemia (11.7 g/dL), thrombocytopenia (107 × 10^9^/L), and a leukocytosis at 5.6 × 10^9^/L (Figure 2D), with 46% of lymphocytes suggestive of hairy cells (Figure 2A). Medullary infiltration persisted with 8% of abnormal cells (Figure 2B). The peripheral karyotype showed a reversal of chromosome 7 and trisomy 5, which are frequent abnormalities in HCL. A splenectomy was performed: splenic histological examination showed infiltration by small cells of B phenotype CD20 positive, CD5 negative, and CD10 negative. The diagnosis between SDRPL and vHCL persisted. The presence of cells with bulky nucleoli however suggested the diagnosis of vHCL. Splenectomy corrected thrombocytopenia and anemia. Four years later, the patient's condition was stable. High‐throughput sequencing analyses show no mutation of the *BRAF* gene but the presence of a *KDM6A* gene mutation.^6^ Sequencing of the variable part of the immunoglobulin heavy chains showed a non‐mutated *IGHV* profile VH4‐34.

## 4 DISCUSSION

The presence of hairy cells in a blood smear could suggest a diagnosis of cHCL, vHCL, SMZL, or SDRPL.^5^ The differences between the four entities are summarized in Table 1. Patients with cHCL have a lower mean age at diagnosis and often have pancytopenia associated with monocytopenia. In cHCL, the villi are not polar, and the nucleoli are not or barely visible. The *BRAF* ^V600E^ mutation is present in more than 80% of cases.^7^ In most cases, patients with vHCL have hyperlymphocytosis with hairy cells with a clearly defined nucleus.^8^ The *BRAF* ^V600E ^mutation is absent and *MAP2K1* gene mutations are observed in one‐third of the cases.^3^ In SMZL,^9^, ^10^ lymphoid cells are often pleomorphic; there is a variable percentage of lymphocytes with polar villi and a little or no visible nucleolus. A monoclonal component and autoimmune manifestations can be observed. In SDRPL,^11^, ^12^, ^13^, ^14^ cytopenias are rare. The villi of the lymphocytes are non‐polar and the nucleoli are absent. Atypical expression of cyclin D3 is also present in tumor cells.^15^ The immunological score of HCL,^2^ based on the expression of CD25, CD11c, CD103, and CD123 is equal to 4 or 3 in the vast majority of cases of cHCL. It is <3 in other hairy cell proliferations. Splenectomy, if it can be performed, shows a characteristic nodular infiltration in SMZL.^16^

**Table 1:** Clinicopathological, biological and treatment comparison among the different diagnoses: SDRPL, SMZL, cHCL, and vHCL

	cHCL	vHCL	SDRPL	SMZL
Average age (y)	55	70	70	70
Splenomegaly	Yes	Yes	Yes	Yes
Lymphocytosis	Low	High	Moderate	Moderate
Anemia/thrombocytopenia	Present	Present	Uncommon	Present
Monocytopenia	Present	Absent	Absent	Absent
Cytological aspect				
Villi	Circumferential	Circumferential	Circumferential, broad based	Polar
Nucleus	Round, oval, or bean‐shaped	Round or oval, sometimes bilobed	Round, sometimes eccentrically placed	Round
Nucleoli	Absent or inconspicuous	Prominent	Absent or inconspicuous	Inconspicuous
Spleen histology	Diffuse	Diffuse	Diffuse	Nodular
Immunophenotype				
CD11c	+	+	+ (50%)	Weak (39%)
CD103	+	+	+ (10%)	+ (40%)
CD123	+	−	−	−
CD25	+	−	+ (25%)	+ (44%)
Annexin A1	+	−	−	−
IGHV	IGHV3‐23 21%, IGHV4‐34 10%, IGHV3‐30 8%	IGHV4‐34 36%	IGHV3‐23, IGHV4‐34	IGHV1‐2 25%, IGHV4‐34 13%, IGHV3‐23 8%
Genetic mutation	*BRAF^V600E^* (>90%)^7^	*MAP2K1* 3 (30%‐40%)	*CCND3* 14, 15 (21%‐24%), *BCOR* 14 (24%)	*NOTCH2* (10%‐25%), *KLF2* (20%‐40%), Epigenetic regulators (40%)^9^
Associated structural abnormality	5 abnormalities del13q, del7q (uncommon)	del17p	Uncommon: del7q, trisomy 18, del17p	del7q; trisomy 3, 12, 18
Treatment	PNA R‐PNA, BRAF inhibitor MP, RB, ibrutinib, splenectomy^17^	R‐PNA MP, ibrutinib, splenectomy^17^	Splenectomy^12^	Splenectomy, R‐chemotherapy (R‐CHOP, RB, RFC…)^10^

Abbreviations: B, bendamustine; cHCL, Hairy Cell Leukemia; CHOP, cyclophosphamide, doxorubicin, vincristine, prednisone; F, fludarabine; MP, moxetumomab pasudotox; PNA, purine analogs; R, rituximab; SDRPL, Splenic Diffuse Red Pulp Lymphoma; SMZL, Splenic Marginal Zone Lymphoma; vHCL, Variant Form of Hairy Cell Leukemia.

In both patients, we retained the diagnosis of vHCL. Splenomegaly, present in both patients, is observed in 85% of cases of vHCL. The pulmonary involvement with appearance of cystic interstitial pneumonitis of the first patient may be associated with vHCL, no other cause having been found. The association between interstitial pneumonitis and hairy cell leukemia was not reported in the literature. The morphology of the lymphocytes in these two patients suggests the diagnosis of vHCL, particularly the presence of a net nucleolus and fine and circumferential villi. Both patients had cytopenias and one of them had significant hyperlymphocytosis. The HCL score at 2 is also consistent with this diagnosis. Splenic histological examination also excludes the diagnosis of SMZL. The absence of a *BRAF* ^V600E^ mutation in both patients and the presence of a *MAP2K1* mutation in the first patient are arguments in favor of the diagnosis of vHCL. Mutations of *KDM6A* have not been described in SDRPL and SMZL.

Given the small number of patients, treatment of vHCL is not yet established.^17^ Purine analogs in monotherapy are not very effective and the first line is often based on the use of purines analogs associated with rituximab. This association was not effective in the first patient who also failed to respond to moxetumomab pasudotox, a new treatment under evaluation.^18^ By contrast, in both patients, splenectomy allowed symptom relief and improved the cytopenias. In the first case, the patient also benefited from ibrutinib. Ibrutinib, an orally available Bruton tyrosine kinase inhibitor (BTK), plays a central role in B‐cell receptor signaling and impacts homing and adhesion of B cells.^19^ Clinical studies on the use of ibrutinib in various non‐Hodgkin's malignant B‐cell lymphomas have shown encouraging results, particularly in chronic lymphocytic leukemia and mantle cell lymphoma.^20^ In vitro studies on HCL have shown that ibrutinib inhibits the survival and proliferation of hairy cells.^21^ A phase II trial is evaluating the use of ibrutinib in patients with relapsed cHCL and vHCL. Preliminary results are encouraging, with partial responses in vHCL with little toxicity after a median follow‐up of 22 months.^22^ After a follow‐up of 4 months, patient 1 was greatly improved by this treatment.

## 5 CONCLUSION

In the age of personalized medicine and increasing treatment choices, it is important to perform an accurate diagnosis to support the patient optimally. The number of cases of vHCL reported in the literature is still low because of the rarity of this hematologic disorder. These two clinical cases illustrate patient profiles that physicians may face in their daily practice. The first patient's profile is that of an aggressive and multiply refractory disease, and the second is of a more indolent disease that responded very well to splenectomy. The two cases represent the heterogeneity of vHCL in terms of clinical presentation as well as management. Despite a short follow‐up period in the case of the first patient, the initial response to treatment remains satisfactory. These cases further underscore that the therapeutic armamentarium for vCHL could be diverse, ranging from ibrutinib to splenectomy.

## CONFLICT OF INTEREST

None declared.

## AUTHOR CONTRIBUTION

MW: collected information on patients and drafted the manuscript. XT, EC: helped in drafting the manuscript and revised contents of the case and discussion. EM, EC, VS, and DN: performed and reviewed the biological vHCL diagnosis.
